# A multivariable prediction model for recovery patterns and time course of symptoms improvement in hemifacial spasm following microvascular decompression

**DOI:** 10.1007/s00701-022-05133-w

**Published:** 2022-02-01

**Authors:** Ahmed Al Menabbawy, Ehab El Refaee, Reem Elwy, Amany A. Salem, Sebastian Lehmann, Marcus Vollmer, Marc Matthes, Steffen Fleck, Jörg Baldauf, Henry W. S. Schroeder

**Affiliations:** 1 Department of Neurosurgery, University Medicine Greifswald, Greifswald, Germany; 2 Department of Neurosurgery, Kasr Alainy School of Medicine, Cairo University, Cairo, Egypt; 3 Department of Public Health, Kasr Alainy School of Medicine, Cairo University, Cairo, Egypt; 4 Institute of Bioinformatics, University Medicine Greifswald, Greifswald, Germany

**Keywords:** Microvascular decompression, Hemifacial spasm, Delayed recovery, Time course of recovery

## Abstract

**Background:**

Microvascular decompression (MVD) success rates exceed 90% in hemifacial spasm (HFS). However, postoperative recovery patterns and durations are variable.

**Objective:**

We aim to study factors that might influence the postoperative patterns and duration needed until final recovery.

**Method:**

Only patients following de-novo MVD with a minimum follow-up of 6 months were included. Overall trend of recovery was modeled. Patients were grouped according to recognizable clinical recovery patterns. Uni- and multivariable analyses were used to identify the factors affecting allocation to the identified patterns and time needed to final recovery.

**Results:**

A total of 323 (92.6%) patients had > 90% symptom improvement, and 269 (77.1%) patients had complete resolution at the last follow-up. The overall trend of recovery showed steep remission within the first 6 months, followed by relapse peaking around 8 months with a second remission ~ 16 months. Five main recovery patterns were identified.

Pattern analysis showed that evident proximal indentation of the facial nerve at root exit zone (REZ), males and facial palsy are associated with earlier recovery at multivariable and univariable levels. anterior inferior cerebellar artery (AICA), AICA/vertebral artery compressions and shorter disease durations are related to immediate resolution of the symptoms only on the univariable level. Time analysis showed that proximal indentation (vs. distal indentation), males and facial palsy witnessed significantly earlier recoveries.

**Conclusion:**

Our main finding is that in contrast to peripheral indentation, proximal indentation of the facial nerve at REZ is associated with earlier recovery. Postoperative facial palsy and AICA compressions are associated with earlier recoveries. We recommend a minimum of 1 year before evaluating the final outcome of MVD for HFS.

## Introduction

Hemifacial spasm (HFS) is a condition affecting the facial nerve usually causing unilateral intermittent and involuntary contractions of facial muscles [2, 7, 9, 33]. This has been attributed to mostly arterial vascular compressions at the root exit zone (REZ) of the facial nerve, increasing its excitability and manifesting as the clinically known facial spasms or contractions that negatively influence the quality of life of these patients.[19, 23, 35]. The exact mechanism of hyperexcitability is still unknown. However, two theories, the central and peripheral theories, have been generally accepted [29, 32]. Microvascular decompression (MVD) is considered an effective curative treatment modality for patients with primary HFS. [7, 33, 36] However, the postoperative patterns of improvement and the time needed for final recovery vary widely among different patients. Furthermore, many factors might influence such patterns or courses of recovery [27, 36]. We aim to develop a multivariable prediction model for the factors affecting the time needed for final symptom resolution and recovery patterns.

## Methods

### Dataset

Patients written consents were obtained preoperatively for using the data for research purposes. Following the approval of our local ethical committee, we retrospectively reviewed our prospectively maintained database/registry of prospectively collected cases of HFS who underwent MVD between 01/2002 and 10/2019. We included only patients that were operated for the first time in our neurosurgical department of the University hospital of University Medicine Greifswald. This study was designed and implemented in accordance with “Transparent reporting of a multivariable prediction model for individual prognosis or diagnosis” (TRIPOD) guidelines statement [8].

**Fig. 1:**
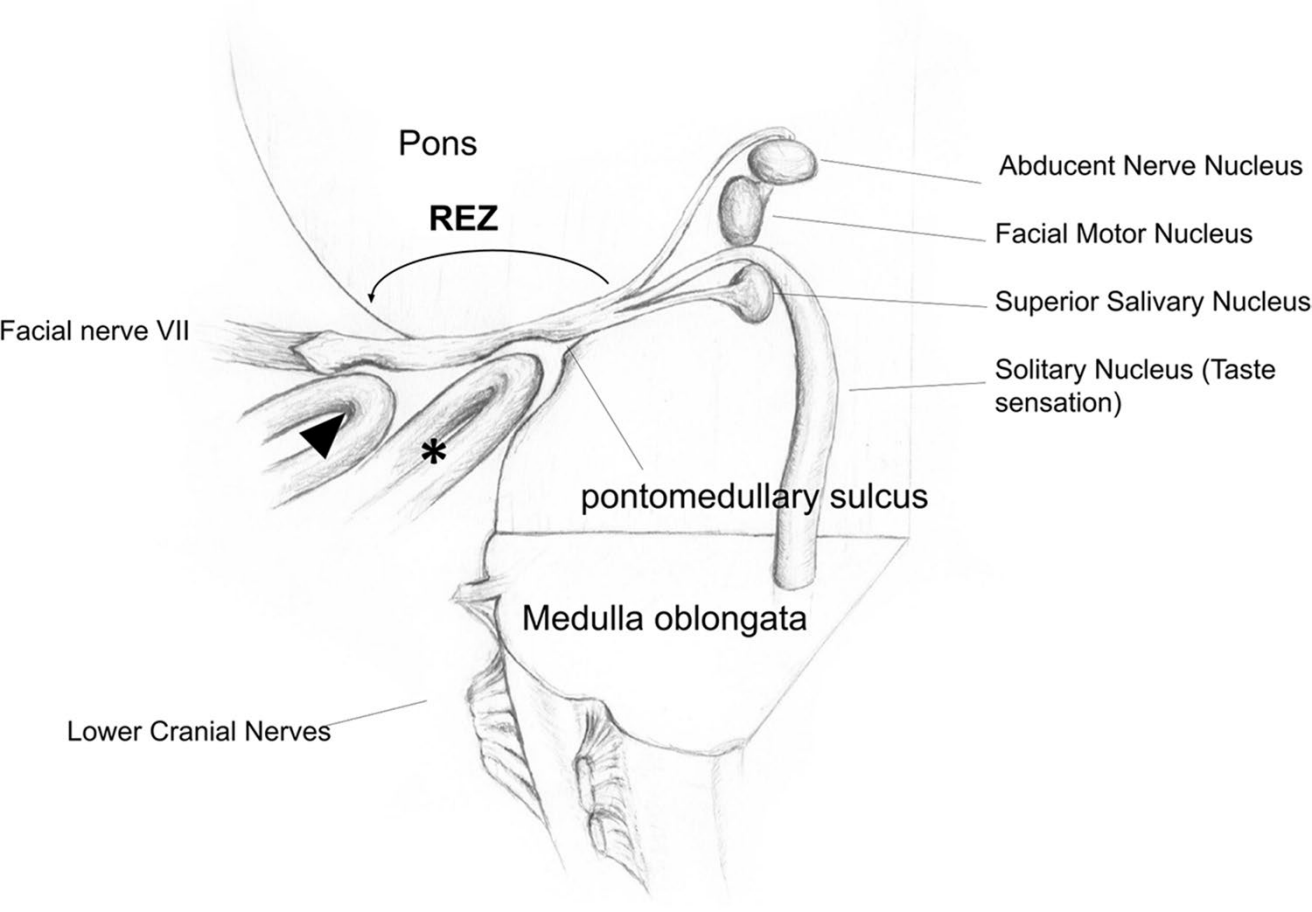
Illustration of different arterial compressions at the root exit zone (REZ) causing proximal indentation/grooving at the brainstem (*) or distal indentation/grooving (arrowhead) just before starting the cisternal part. Facial nerve myelinated fibers shown from proximal to distal with transitional zone between central and peripheral myelin

**Fig. 2:**
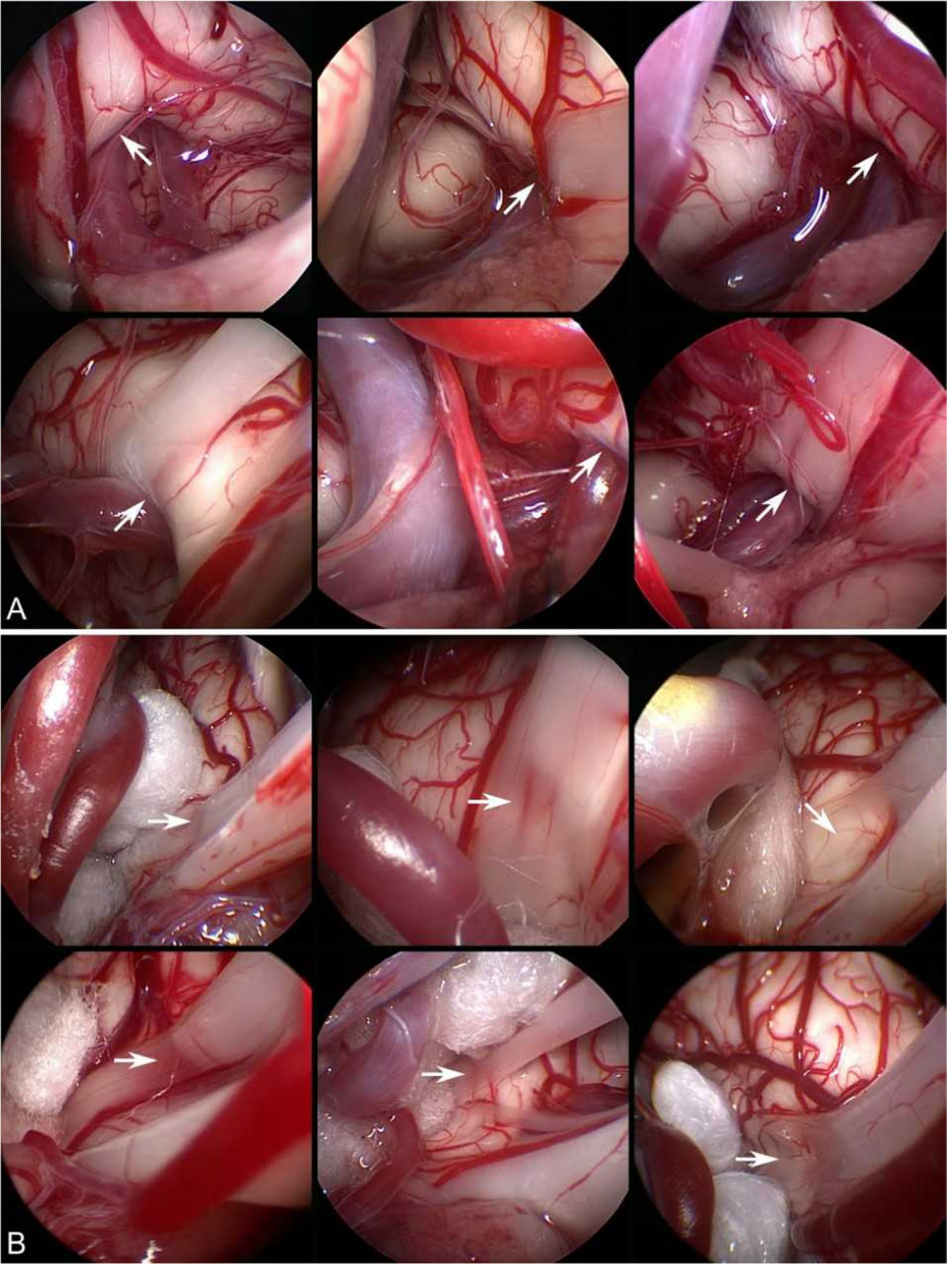
Endoscopic inspection with a 45° endoscope showing grooving/indentation of the facial nerve. **A**: proximal grooving at the brainstem (arrows). **B**: distal grooving where the nerve leaves the brainstem (arrows)

We extracted data on patient demographics, preoperative symptom duration, side, etiology of the compressing vessel(s), type of surgical decompression performed (Interposition with shredded Teflon vs. Transposition with sling retraction), intraoperative evident indentation/grooving of the facial nerve (either “proximal” at the pontomedullary sulcus or “distal” around 5 mm distal to the sulcus just around the beginning of the cisternal part of the nerve (Figs. 1 and 2)).

Furthermore, postoperative development of delayed facial palsy, postoperative adjuvant Botox injections and follow-up duration were noted. We assessed postoperative outcome clinically based on patient reported symptom severity as a percentage of preoperative symptoms at each follow-up where the preoperative condition was considered 100% symptom severity. Exclusion criteria were cases with < 6-month follow-up or non-arterial compression. In patients with more than one surgical procedure for HFS, only data regarding the first operation were included.

### Outcomes and predictors

#### Pattern analysis

Firstly, the overall general recovery pattern of all patients was studied (as percentage of residual symptoms). Five different common clinical patterns of recovery were identified. Finally, we analyzed factors predicting the allocation of patients to each of the identified groups of remission patterns.

#### Time analysis

Our secondary outcome was the final 100% postoperative resolution of the spasms for every individual patient disregarding the pattern of recovery.

### Preoperative imaging

To identify the cause of HFS, careful analysis of the preoperative MR imaging with constructive interference in steady state (3D CISS) and time of flight (3D TOF) angiography sequences was performed.

### Operative technique

All MVDs were performed by the senior author (HWSS) through a lower retrosigmoid craniotomy in supine position under facial electromyography (EMG) and brainstem auditory evoked potential (BAEP) monitoring. Lateral spread response was recorded routinely. After arachnoid dissection, the facial REZ was visualized with a 45° endoscope [12]. The course of the facial nerve was inspected. Depending on the anatomical situation, the decompression was done by interposing shredded Teflon or transposing the vessel using a Gortex or Teflon sling and fixing it to the skull base dura [13]. Every attempt was made to leave the original compression zone free of contact to Teflon [9, 11]. The exact etiology and type of compression as well as visual control of decompression were more precisely determined intraoperatively by endoscopic inspection using a 45° endoscope [12]. Intraoperative determination of facial nerve grooving was noted as well as its position.

### Statistical methods

Analysis was performed using R software version 3.6.3 (R Foundation for Statistical Computing, Vienna, Austria) [40]. Continuous variables were described using mean ± standard deviation (SD) or median with interquartile range (IQR) according to distribution. Categorical variables were described as counts and percentages. The trend of the postoperative course of recovery was estimated using a generalized additive model of the severity of symptoms over time using ggplot2 package version 3.3.3 [43]. To compare the five groups of postoperative remission patterns, multinomial logistic regression was performed using IBM® SPSS® version 22.0 (IBM Corp., Armonk, NY, USA), for which we reported odds ratio (OR), 95% confidence intervals (CI) and *P* values.

Time to final recovery was demonstrated by Kaplan–Meier curves. The difference in time to recovery between different groups was compared using the Log-Rank test. When pairwise comparisons were done, a Benjamini–Hochberg (BH) adjustment was applied. Two uni- and multivariable Cox regression analyses were performed to identify independent factors affecting time to final recovery with reporting of hazard ratios (HR), 95% CI and *P* values. Survival analysis was performed using the survival package version 3.2–7 [40]. For all regression analyses: all a priori selected predictors were entered in multivariable analysis regardless of their significance on the univariable level, and stepwise backward elimination was used to select the model of best fit. Statistical significance was set at a *P* value of < 0.05 (two-tailed).

## Results

### Study population and overall recovery

A total of 379 patients underwent a total of 416 operations. Thirty-seven data entries for the second or third operation for the same patient were excluded from our analysis. Follow-up data could not be obtained in 7 patients. Ten patients had a follow-up < 6 months, and 9 patients were reoperated within the first 6 months following the first operation. Four patients had intraoperatively no evident arterial compression of the nerve (1 venous compression, 2 arachnoid band strictures and 1 patient with no compression who had synkinesis after facial palsy, but no hemifacial spasm). Finally, 349 data entries of de-novo MVD were included. The mean (SD) follow-up was 54.6 (35.9) months.

A total of 323 patients (92.6%) had a satisfactory outcome with at least 90% resolution of the symptoms, while 308 patients (88.3%) showed at some point 100% complete resolution of the symptoms along the postoperative course; 269 (77.1%) patients had complete resolution of the symptoms at the last follow-up. Only 3 patients (0.9%) showed no improvement at all after surgery. General characteristics of the study population and the follow-up period are tabulated in Table 1. A total of 246 patients (70.5%) showed significant intraoperative arterial compressions without grooving of the nerve, while 103 patients had visible indentation/grooving of the nerve at the site of compression; 52 patients (14.9%) with proximal grooving (Fig. 2A) and 51 patients (14.6%) with distal grooving (Fig. 2B). Postoperative facial nerve weakness occurred in 32 cases (9.2%). Six patients (1.7%) out of these had an immediate facial nerve weakness, while 26 patients (7.4%) experienced a delayed facial nerve weakness occurring usually 10 days after surgery.

**Table 1:** Summary of predictor risk factors by improvement pattern

Dependent: Improvement pattern		Group 1 (Immediate Resolution)	Group 2 (Gradual Resolution)	Group 3 (Eventual Resolution)	Group 4 (Eventual Relapse)	Group 5 (No full Resolution)	Total
Total N (%)		129 (37.0)	86 (24.6)	54 (15.5)	39 (11.2)	41 (11.7)	349
Follow up (months)	Mean (SD)	51.2 (35.9)	62.5 (33.7)	68.7 (35.4)	54.9 (35.3)	30.2 (28.8)	54.6 (35.9)
Age (years)	Mean (SD)	56.1 (12.3)	53.5 (11.4)	55.8 (12.7)	55.6 (11.3)	54.1 (12.3)	55.1 (12.1)
Sex	Female	61 (47.3)	47 (54.7)	42 (77.8)	30 (76.9)	29 (70.7)	209 (59.9)
	Male	68 (52.7)	39 (45.3)	12 (22.2)	9 (23.1)	12 (29.3)	140 (40.1)
Symptom Duration (years)	Median (IQR)	6.0 (7.0)	7.0 (6.0)	5.0 (4.8)	8.0 (6.0)	7.0 (5.0)	7.0 (6.0)
Side	Left	80 (62.0)	50 (58.1)	27 (50.0)	29 (74.4)	22 (53.7)	208 (59.6)
	Right	49 (38.0)	36 (41.9)	27 (50.0)	10 (25.6)	19 (46.3)	141 (40.4)
Etiology	PICA	38 (29.5)	41 (47.7)	15 (27.8)	13 (33.3)	15 (36.6)	122 (35.0)
	AICA	35 (27.1)	14 (16.3)	16 (29.6)	17 (43.6)	10 (24.4)	92 (26.4)
	AICA PICA	11 (8.5)	8 (9.3)	5 (9.3)	2 (5.1)	4 (9.8)	30 (8.6)
	AICA PICA VA	8 (6.2)	4 (4.7)	0 (0.0)	0 (0.0)	1 (2.4)	13 (3.7)
	AICA VA	19 (14.7)	7 (8.1)	9 (16.7)	3 (7.7)	3 (7.3)	41 (11.7)
	PICA VA	14 (10.9)	9 (10.5)	7 (13.0)	4 (10.3)	4 (9.8)	38 (10.9)
	VA	4 (3.1)	3 (3.5)	2 (3.7)	0 (0.0)	4 (9.8)	13 (3.7)
Decompression	Teflon	119 (92.2)	80 (93.0)	51 (94.4)	35 (89.7)	38 (92.7)	323 (92.6)
	Sling	10 (7.8)	6 (7.0)	3 (5.6)	4 (10.3)	3 (7.3)	26 (7.4)
Grooving	No	96 (74.4)	51 (59.3)	44 (81.5)	32 (82.1)	23 (56.1)	246 (70.5)
	Brainstem	20 (15.5)	20 (23.3)	5 (9.3)	3 (7.7)	4 (9.8)	52 (14.9)
	Peripheral	13 (10.1)	15 (17.4)	5 (9.3)	4 (10.3)	14 (34.1)	51 (14.6)
Facial Palsy	No	109 (84.5)	83 (96.5)	48 (88.9)	36 (92.3)	41 (100.0)	317 (90.8)
	Yes	20 (15.5)	3 (3.5)	6 (11.1)	3 (7.7)	0 (0.0)	32 (9.2)

### General trend of recovery and different patterns within the study population

**Fig. 3:**
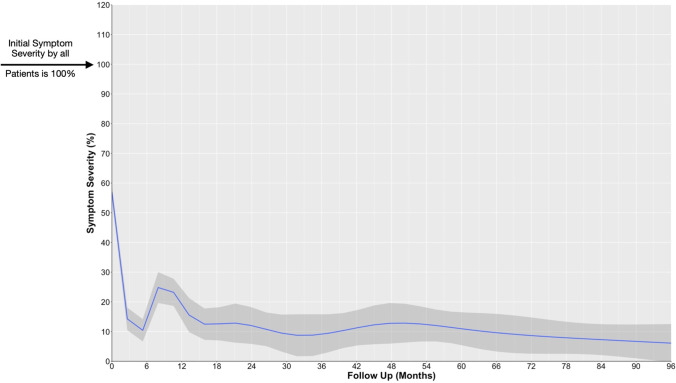
Overall trend of postoperative symptoms resolution following MVD in HFS in our patients. Initial symptom severity in all patients was 100%. Follow-up beyond 8 years is not depicted on this graph

**Fig. 4:**
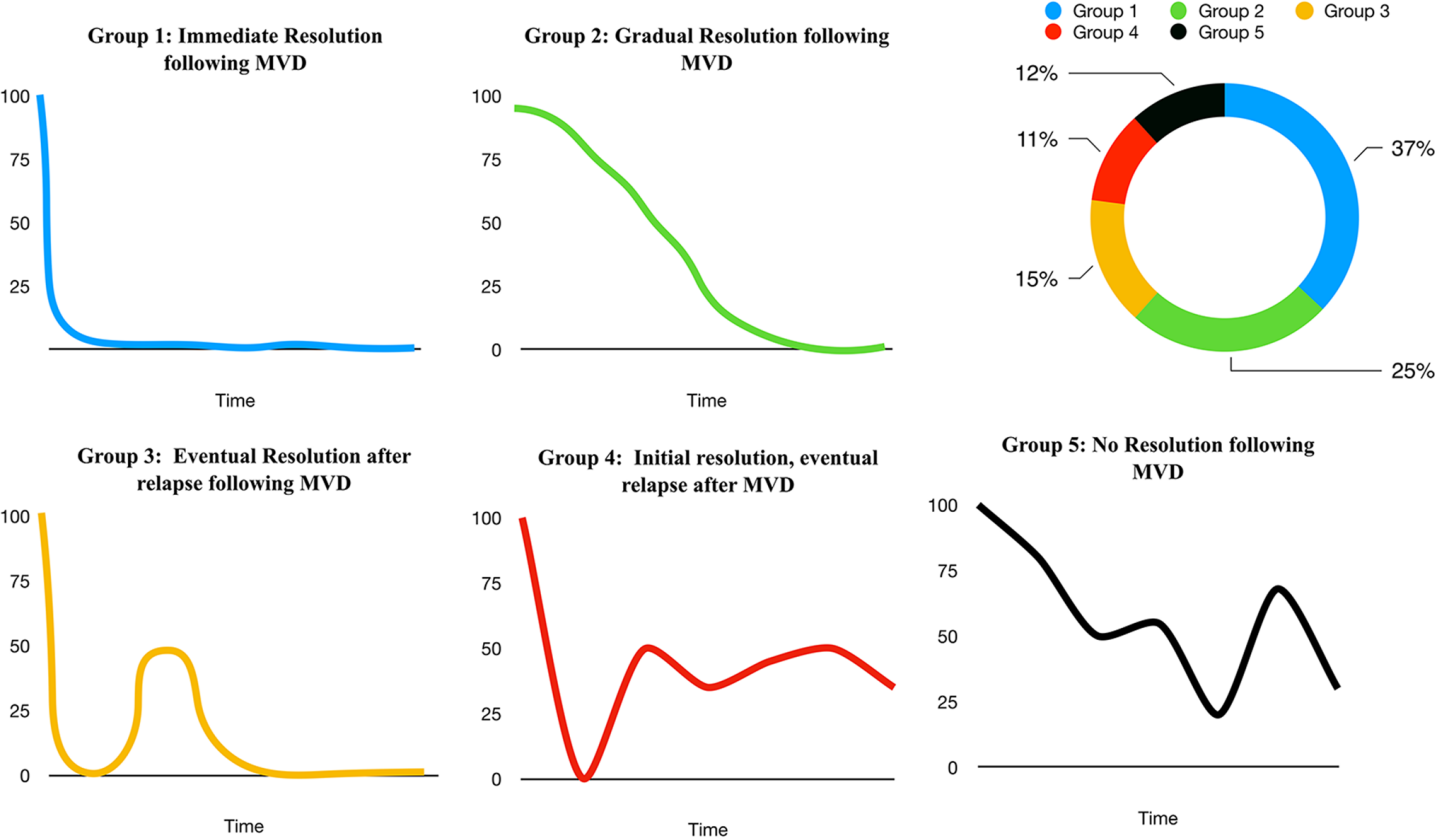
Illustration sketch of the different patterns of symptoms resolution following MVD in HFS. The five groups: Group 1: immediate full resolution, Group 2: gradual full resolution, Group 3: eventual resolution, Group 4: initial full resolution with relapse(s) **without** final resolution and Group 5: improvement without full resolution and pie chart showing percentage distribution of each group in our patients

The overall postoperative recovery of all patients was plotted against time as illustrated in Fig. 3. A steep remission is witnessed within the first 6 months after surgery followed by a relapse peaking at ~ 8 months with a second improvement at 13–16 months after surgery; 90.2% of the patients who demonstrated postoperative complete resolution of the symptoms reported it within 16 months after surgery, and 88% of them experienced full resolution within 12 months. We identified 5 main clinical patterns (Fig. 4 and Table 1):

- Group 1: Immediate full resolution 129/349 (36.96%)
- Group 2: Gradual full resolution 86/349 (24.64%)
- Group 3: Initial full resolution with relapse(s) and final resolution “Eventual Resolution” 54/349 (15.47%)
- Group 4: Initial full resolution with relapse(s) **without** final resolution 39/349 (11.17%)
- Group 5: Improvement without full resolution at any point 41/349 (11.75%)

Resolution is defined as 100% disappearance of the symptoms. Despite residual symptoms in groups 4 and 5, 54/80 patients (67.5%) had > 90% improvement at the last follow-up.

On the univariable level, patients with anterior inferior cerebellar artery AICA (OR: 0.37; CI 0.17–0.79), or AICA/vertebral artery (VA) compression (OR: 0.34; CI 0.13–0.90), as well as patients who developed postoperative facial palsy (OR: 0.2; CI 0.06–0.69) had lower odds of having a delayed (group 2) versus immediate resolution (group 1). Male patients (OR: 0.26; CI 0.12–0.53), or patients with a longer symptom duration (OR: 0.92; CI 0.85–0.99) had lower odds of having a relapse before final resolution (group 3) in comparison to immediate resolution (group 1). Male patients also had lower odds of having final residual spasms (group 4) (OR: 0.27; CI 0.12–0.61) versus immediate resolution (group 1). Male gender (OR: 0.37; CI 0.17–0.79) had also lower odds of allocation to group 5 in comparison to group 1, while peripheral grooving of the facial nerve (OR: 4.5; CI 1.86–10.85) predicted incomplete resolution of symptoms (group 5) compared to group 1.

In the multivariable analysis, statistically significant independent predictors of allocation to one of the five defined remission patterns were sex, location of vessel induced grooving and postoperative facial palsy. Compared to group 1, male patients were less likely to be allocated to group 3 (OR: 0.25; CI 0.12–0.54), group 4 (OR: 0.28; CI 0.12–0.65) or group 5 (OR: 0.39; CI 0.18–0.87). Peripheral grooving increased the odds of patient allocation to group 2 (OR: 2.32; Ci 1.01–5.35) or group 5 (OR: 5.32; CI 2.12–13.40), whereas the development of postoperative facial palsy decreased the odds of patient allocation to group 2 (OR: 0.2; CI 0.06–0.7) in comparison to group 1. The performed technique of decompression (interposition vs. transposition) and the remaining tested factors did not show statistical significance on influencing the pattern of recovery.

### Factors affecting time to final recovery

**Fig. 5:**
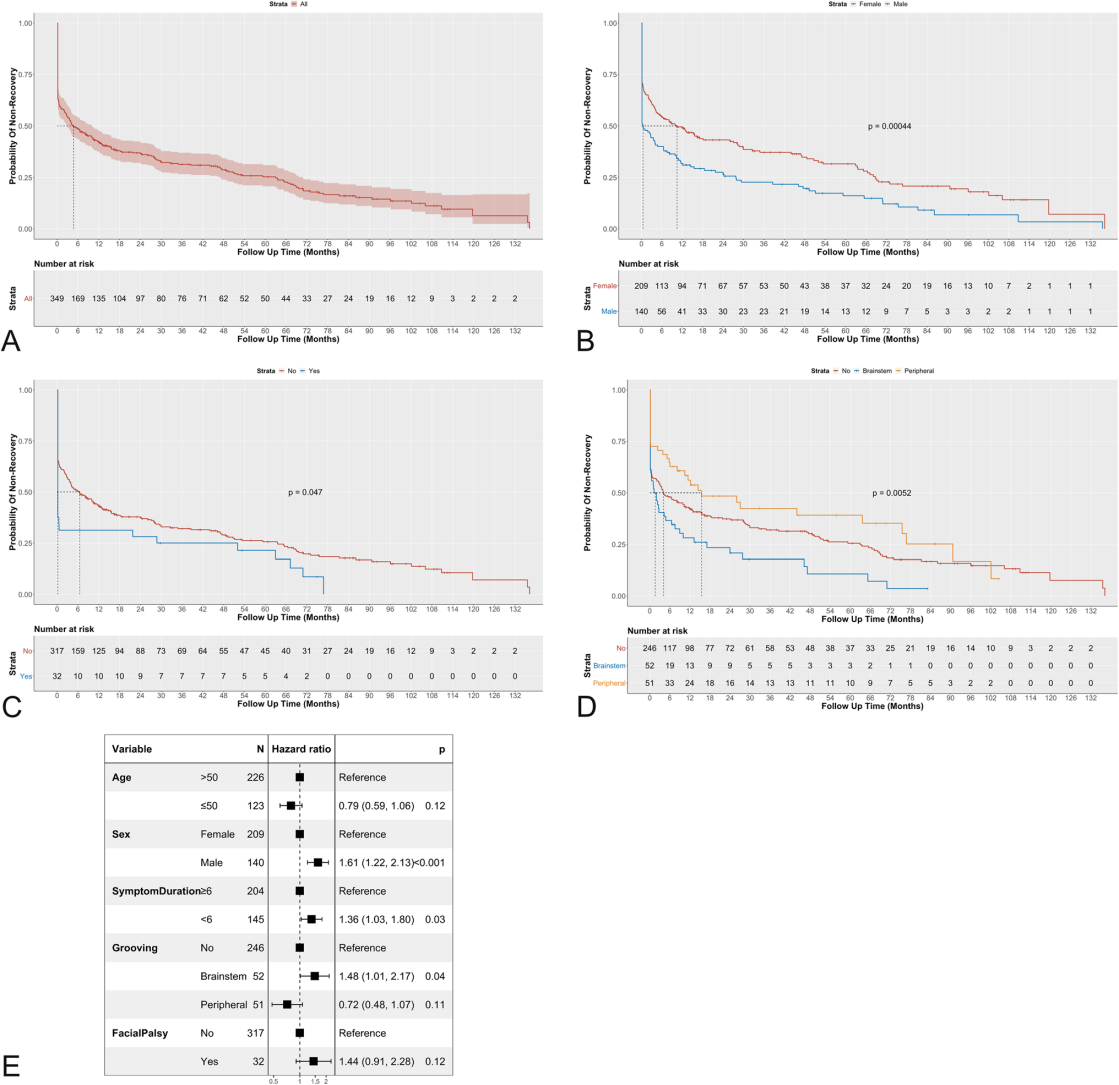
Kaplan–Meier curves illustrate time to final resolution of the spasms following MVD for all patients and after stratification according to individual significant variables with comparison of the strata using the log rank test. **A**: for all patients without comparing any factors, **B**: sex (males and females), **C**: facial palsy yes/no, **D**: presence/absence of indentation/grooving of the nerve and its location and **E**: Forrest plot for independent factors affecting time to final recovery showing significance of sex, disease duration and location of the facial nerve grooving

Eventual complete resolution of symptoms regardless of postoperative course fluctuations was reached in 269 (77.1%) patients occurring in 50% at a median of 142 days (4.7 months) (95%CI 83–300) after surgery. A significant difference in time to final recovery according to sex (*p* < 0.001), development of postoperative facial palsy (*p* = 0.047) and the location of vessel induced grooving (*p* = 0.005) was shown (Fig. 5). Pairwise comparison showed a significantly shorter time to recovery with proximal nerve grooving (median: 49 days; 95%CI 5–227) compared to no grooving (median: 122.5; 95%CI 63–325; *p* = 0.02), and to distal grooving (median: 465 95%CI 246–2303; *p* = 0.002). Statistically significant factors affecting time to final recovery are shown in Fig. 5. Results of the multivariable and univariable analysis of risk factors for final recovery for patients with a minimum follow-up of 6 months as well as those with a minimum follow-up of 12 months are tabulated in Table 2.

**Table 2:** Multivariable and Univariable Analysis of Risk Factors for Final Recovery

		Follow up ≥ 6 months			Follow up ≥ 1 year		
Dependent: Final Recovery		all	HR (univariable)	HR (multivariable reduced)^b^	all	HR (univariable)	HR (multivariable reduced)^a^
Age (years)	> 50	226 (64.8)	-	-	206 (64.6)	-	-
	≤ 50	123 (35.2)	0.86 (0.65–1.14, *p* = 0.308)	0.79 (0.59–1.06, *p* = 0.116)	113 (35.4)	0.87 (0.66–1.17, *p* = 0.363)	0.80 (0.59–1.08, *p* = 0.151)
Sex	Female	209 (59.9)	-	-	190 (59.6)	-	-
	Male	140 (40.1)	1.61 (1.23–2.11, ***p* < 0.001**)	1.61 (1.22–2.13, ***p* = 0.001**)	129 (40.4)	1.65 (1.25–2.17, ***p* < 0.001**)	1.63 (1.22–2.17, ***p* = 0.001**)
Symptom Duration (years)	≥ 6	204 (58.5)	-	-	184 (57.7)	-	-
	< 6	145 (41.5)	1.26 (0.97–1.65, *p* = 0.086)	1.36 (1.03–1.80, ***p* = 0.028**)	135 (42.3)	1.26 (0.95–1.66, *p* = 0.105)	1.35 (1.02–1.80, ***p* = 0.038**)
Side	Left	208 (59.6)	-	-	191 (59.9)	-	-
	Right	141 (40.4)	0.98 (0.75–1.29, *p* = 0.906)	-	128 (40.1)	0.94 (0.71–1.24, *p* = 0.642)	-
Etiology	PICA	122 (35.0)	-	-	114 (35.7)	-	-
	AICA	92 (26.4)	0.92 (0.65–1.30, *p* = 0.632)	-	84 (26.3)	0.93 (0.65–1.33, *p* = 0.687)	-
	AICA PICA	30 (8.6)	1.57 (0.93–2.64, *p* = 0.090)	-	24 (7.5)	1.83 (1.05–3.19, *p* = 0.034)	-
	AICA PICA VA	13 (3.7)	1.93 (0.95–3.91, *p* = 0.067)	-	12 (3.8)	1.87 (0.90–3.88, *p* = 0.094)	-
	AICA VA	41 (11.7)	1.18 (0.77–1.81, *p* = 0.455)	-	39 (12.2)	1.16 (0.75–1.80, *p* = 0.512)	-
	PICA VA	38 (10.9)	1.25 (0.79–1.98, *p* = 0.337)	-	35 (11.0)	1.42 (0.89–2.27, *p* = 0.139)	-
	VA	13 (3.7)	0.86 (0.41–1.80, *p* = 0.686)	-	11 (3.4)	0.88 (0.40–1.92, *p* = 0.741)	-
Decompression	Teflon	323 (92.6)	-	-	296 (92.8)	-	-
	Sling	26 (7.4)	0.79 (0.47–1.30, *p* = 0.349)	-	23 (7.2)	0.75 (0.44–1.27, *p* = 0.285)	-
Grooving	No	246 (70.5)	-	-	228 (71.5)	-	-
	Brainstem	52 (14.9)	1.63 (1.12–2.39, ***p* = 0.011**)	1.48 (1.01–2.17, ***p* = 0.044**)	46 (14.4)	1.65 (1.11–2.45, ***p* = 0.013**)	1.48 (1.00–2.21, *p* = 0.052)
	Peripheral	51 (14.6)	0.73 (0.49–1.09, p = 0.129)	0.72 (0.48–1.07, *p* = 0.105)	45 (14.1)	0.77 (0.51–1.17, p = 0.217)	0.75 (0.50–1.14, *p* = 0.180)
Facial Palsy	No	317 (90.8)	-	-	287 (90.0)	-	-
	Yes	32 (9.2)	1.56 (1.00–2.43, ***p* = 0.048**)	1.44 (0.91–2.28, *p* = 0.122)	32 (10.0)	1.58 (1.01–2.46, ***p* = 0.045**)	1.45 (0.91–2.31, *p* = 0.115)

^b^Number in dataframe = 349, Number in model = 349, Missing = 0, Number of events = 269, Concordance = 0.619 (SE = 0.022), R-squared = 0.080( Max possible = 0.993), Likelihood ratio test = 29.252 (df = 6, *p* = 0.000)

^a^Number in dataframe = 319, Number in model = 319, Missing = 0, Number of events = 251, Concordance = 0.617 (SE = 0.022), R-squared = 0.081( Max possible = 0.993), Likelihood ratio test = 27.016 (df = 6, *p* = 0.000)

## Discussion

Several studies investigated the predicting factors influencing the outcome following MVD in HFS patients [6, 17, 18, 23, 39]. Fewer studies analyzed the course of symptoms improvement and patterns of recovery following MVD attributing delayed resolution of the spasms to various predicting factors [18, 24, 27, 32, 34]. Our time analysis addressed each patient individually while the trend analysis addressed the different trend groups.

### Significance of facial nerve indentation/grooving

The main distinctive finding of our study is showing that the location of facial nerve grooving is a prognostic factor regarding the outcome. Distal indentation is associated with delayed recovery and poor outcome, while proximal grooving correlates with earlier recovery and better outcomes. Some authors reported that intraoperative evident indentation of the facial nerve is a good prognostic factor for symptom improvement following MVD for HFS [21, 26], while others considered it a poor prognostic factor [20]. However, the exact position of that indentation was not clearly mentioned or identified. Studying the microscopic anatomy of the REZ and proximal part of the facial nerve is pivotal to interpret such differences regarding the outcome.

Firstly, not all fibers leave the brainstem at the same point. In fact, they exit over a segment (REZ). Moreover, the fibers continue to be covered by central myelin till the end of the transitional zone (Redlich-Obersteiner zone) which continues till the proximal part of the nerve root. [5, 31, 41, 44]

Secondly, it is agreed upon that compressions peripheral to the transitional zone of myelin and covered only by peripheral myelin are usually insignificant [8, 14]. However, it is also important to note that the transition zone length between central and peripheral myelin is variable among the different patients [27, 30].

We identified indentation of the nerve in 103 patients (52 proximal and 51 distal). Our analysis of time needed until recovery showed strong statistical significance. On one hand, proximal indentation is associated with earlier final recovery, and on the other hand, distal indentation was a strong predictor for failure of complete resolution (group 5) and longer time for recovery.

Delayed cure seems logical if considering that primary HFS is due to hyperactivity in facial nerve due to chronic irritation caused by the vascular compression. The significant difference in outcome between proximal and distal grooving may be attributed to the sudden alleviation of the facial nerve nucleus irritation following proximal decompression or due to the complexity of the regeneration process at the distal transitional zone between central and peripheral myelin. Our described distal indentation usually took place in the transitional zone, and this might be the reason why recovery takes longer in these patients. More peripherally located compressions beyond the transitional zone in areas solely myelinated with peripheral myelin show high resistance to vascular compressions and usually do not cause HFS. [10, 15, 16]

### When to re-operate?

Some authors reported that failure to improve within the first postoperative week warrants reoperation.[37], Lee et al.[24] suggested that 6 months is the minimum period to wait before deciding for the outcome of the MVD as they showed similar outcomes at 6 and 9 months to the outcomes at 12 months. In our series, a remarkable recovery is also witnessed within the first 6 months (Fig. 3) after surgery, which supports the results of Lee et al.[24] However, a general partial relapse of the symptoms follows around 8^th^ month with secondary remission around 13th month postoperatively. Therefore, monitoring the postoperative course would be helpful as it would reflect the success of the operation largely. A relapse of the symptoms should be met with some patience without rushing directly to reoperation. By failure of considerable improvement within 12 months, reoperation should be considered.

We disagree with authors who suggested that 3 months of follow-up is enough to evaluate the outcome following MVD for HFS [21, 34]. However, we agree with authors who suggested waiting for longer periods of at least 1 year. [24, 33]

### Does sex matter?

Although males and females did not differ regarding the overall final outcome, gender showed surprisingly significant difference regarding the pattern and time course of recovery. Males are more likely to show earlier improvement and less likely to develop relapses. Most of the previous studies and reports including our own previous studies did not show significant difference regarding sex on the final outcome. [28, 41] However, in few studies males’ privilege was reported regarding long-term success and lower recurrence rates following MVD in comparison to females. [3, 4] We cannot explicitly explain these results. However, this might be related to the partial subjectivity of the assessment as outcomes depend also on the patient’s self-assessment of improvement and some studies reported differences between males and females regarding describing and reporting symptoms in general. In such studies, it has been stated that females are more likely to describe symptoms more often and precisely than men. This might explain why women reported more relapses and delayed recoveries in comparison to men despite the fact that the final outcome did not differ significantly. [1, 22, 38] Therefore, we recommend in the near future developing new more objective tools to assess spasm-intensity and frequency.

### Significance of the compressing vessel

Compressions caused by AICA and complex compression with AICA and VA were associated with immediate resolution rather than a delayed recovery. Some authors showed similar results to our results, while other authors declared also better outcomes in cases of compressions caused by PICA rather than other vessels [27, 30].

### Duration of the disease and postoperative course

Longer disease durations have been usually related to poor outcomes [27, 30]. In our study, shorter disease history less than 6 years was associated with earlier final recovery, but it is not significant on the long-term results. Obviously, prolonged compression might contribute to more structural changes of the nerve and so more time for regeneration.

### Facial palsy: friend or enemy?

Delayed facial nerve weakness should be distinguished from the immediate postoperative weakness. In our study, we had 32 patients (9.2%) with postoperative facial palsy. 6 of whom (1.7%) had an immediate postoperative facial nerve weakness which we relate to intraoperative manipulations. The other 26 patients experienced a delayed weakness in which the exact etiology is still unclear. Interestingly, some studies showed that postoperative delayed facial nerve palsy following MVD is significantly associated with a more favorable outcome after MVD for HFS in both the short and long terms [24, 25]. Our results support this. However, we still consider it also a complication despite its good prognostic value.

### Limitations

We excluded patients with only venous and/or arachnoid band compression, which might also play a role influencing the postoperative recovery. In our series, we had only one patient with pure venous compression who is completely spasm-free immediately after the surgery. Some studies highlighted that arachnoid bands and venous compressions, although rare, may correlate with bad outcomes [14, 30, 42].

## Conclusion

We advise to wait at least 12 months before assessing the outcome of MVD in HFS. Proximal indentation of the facial nerve at the REZ as well as postoperative facial palsy is associated with earlier recovery. On the other hand, distal indentation is associated with relatively worse outcome and longer time for recovery.
